# Key normative, legal, and policy considerations for supporting pregnant and postpartum adolescents in high HIV-burden settings: a critical analysis

**DOI:** 10.1080/26410397.2023.2249696

**Published:** 2023-09-15

**Authors:** Christina A. Laurenzi, Elona Toska, Renata Tallarico, Lorraine Sherr, Kathryn J. Steventon Roberts, Maja Hansen, Janke Tolmay, Janina Jochim, Wole Ameyan, Rachel Yates

**Affiliations:** a Senior Researcher, Institute for Life Course Health Research, Department of Global Health, Faculty of Medicine and Health Sciences, Stellenbosch University, Tygerberg, South Africa.; b Associate Professor, Centre for Social Science Research, Department of Sociology, University of Cape Town, Rondebosch, South Africa; Co-director, Accelerate Hub, University of Cape Town, Rondebosch, South Africa; Associate Professor, Department of Social Policy and Intervention, Oxford University, Oxford, United Kingdom; c Youth Team Lead and SYP Regional Coordinator, United Nations Population Fund, Eastern and Southern Regional Office, Johannesburg, South Africa; d Professor, Clinical and Health Psychology, University College London, London, United Kingdom; e Postdoctoral Researcher, Department of Social Policy and Intervention, University of Oxford, Oxford, United Kingdom; Postgraduate Researcher, Institute for Global Health, University College London, London, United Kingdom; f Technical Advisor, Gender Equality, United Nations Population Fund, Dar es Salaam, Tanzania; g Quantitative Research Assistant, Accelerate Hub, Centre for Social Science Research, University of Cape Town, Rondebosch, South Africa; h Postdoctoral Research Officer, Department of Social Policy and Intervention, University of Oxford, Oxford, United Kingdom; i Technical Officer, Adolescent HIV, Global HIV Hepatitis and Sexually Transmitted Infections Programmes, World Health Organization, Geneva, Switzerland; j Strategic Advocacy Lead, Accelerate Hub, University of Oxford, Oxford, United Kingdom

**Keywords:** Adolescent motherhood, HIV, child marriage, violence, healthcare access, sexual and reproductive health and rights, gender inequalities, gender norms, social protection

## Abstract

Rates of adolescent pregnancy within sub-Saharan Africa are increasing. Adolescent mothers ages 10–19 years face a distinct set of risks to their own and their children’s health, compounded by many economic, social, and epidemiological challenges, such as living with HIV. In navigating this complex developmental period, many adolescent mothers face structural barriers impeding safe transitions to adulthood and motherhood. Drawing on existing literature and emerging data, we outline three normative, legal, and policy issues – violence and gender inequity, access to sexual and reproductive health services, and access to social and structural supports – which affect the health, wellbeing and development of adolescent mothers and their children. We also highlight emergent evidence about programming and policy changes that can better support adolescent mothers and their children. These key proposed responses include removing barriers to SRH and HIV service integration; ensuring implementation of return-to-school policies; and extending social protection systems to cater for adolescent mothers. Despite ongoing global crises and shifts in funding priorities, these normative, legal, and policy considerations remain critical to safeguard the health and wellbeing of adolescent mothers and their children.

## Résumé

Les taux de grossesse chez les adolescentes sont en augmentation en Afrique subsaharienne. Les mères adolescentes âgées de 10 à 19 ans sont confrontées à un ensemble distinct de risques pour leur santé et celle de leurs enfants, des risques aggravés par de nombreuses difficultés économiques, sociales et épidémiologiques, comme vivre avec le VIH. Au cours de cette période de développement complexe, beaucoup de mères adolescentes se heurtent à des obstacles structurels qui les empêchent de faire la transition vers l’âge adulte et la maternité en toute sécurité. Nous fondant sur les publications existantes et les données émergentes, nous dégageons trois problèmes normatifs, juridiques et politiques – la violence et l’inégalité entre les sexes, l’accès aux services de santé sexuelle et reproductive, et l’accès aux soutiens sociaux et structurels – qui influent sur la santé, le bien-être et le développement des mères adolescentes et de leurs enfants. Nous mettons aussi en avant les données émergentes sur les changements de programmes et de politiques qui peuvent mieux soutenir les mères adolescentes et leurs enfants. Ces mesures clés proposées comprennent la levée des obstacles à l’intégration des services de SSR et de prise en charge du VIH ; la mise en œuvre de politiques de retour à l’école ; et l’élargissement des systèmes de protection sociale pour répondre aux besoins des mères adolescentes. En dépit des crises mondiales actuelles et des réorientations des priorités de financement, ces considérations normatives, juridiques et politiques restent capitales pour protéger la santé et le bien-être des mères adolescentes et de leurs enfants.

## Resumen

En África subsahariana, las tasas de embarazo en la adolescencia están en alza. Las madres adolescentes de 10 a 19 años enfrentan riesgos específicos a su salud y a la de sus hijos, agravados por muchos retos económicos, sociales y epidemiológicos, como vivir con VIH. Al navegar este complejo período de desarrollo, muchas madres adolescentes enfrentan barreras estructurales que impiden transiciones seguras a la adultez y la maternidad. A raíz de la literatura disponible y datos emergentes, identificamos tres asuntos normativos, legislativos y políticos –violencia y desigualdad de género, acceso a servicios de salud sexual y reproductiva y acceso a apoyo social y estructural— que afectan la salud, el bienestar y el desarrollo de las madres adolescentes y sus hijos. Además, destacamos evidencia emergente sobre los retos de programas y políticas que puedan apoyar mejor a las madres adolescentes y a sus hijos. Entre las respuestas propuestas clave figuran: eliminar las barreras a la integración de servicios de SSR y VIH; garantizar la aplicación de políticas relativas al regreso a la escuela; y extender los sistemas de protección social para atender a madres adolescentes. A pesar de las continuas crisis mundiales y cambios en las prioridades de financiamiento, estas consideraciones normativas, legislativas y políticas continúan siendo fundamentales para salvaguardar la salud y el bienestar de madres adolescentes y sus hijos.

## Introduction

The number of births to mothers younger than 20 years old is projected to rise by 2030, and new data show that one in three young women in low- and middle-income countries (LMICs) start childbearing in adolescence.^1–3^ Girls who become mothers by the age of 19, here referred to as adolescent mothers, face a distinct set of risks to their own health and to their child’s health.^4,5^ Complications in pregnancy and childbirth are the leading cause of death for adolescent girls ages 15–19 years in LMICs.^6^ Adolescents who do give birth have higher risks of adverse birth outcomes, including eclampsia/pre-eclampsia, preterm birth, and low birth weight.^7, 8^ Additionally, worldwide, at least 10 million of the annual 21 million pregnancies to adolescent girls ages 15–19 are unplanned and unintended^3^ – and as many as 95% in emerging data from South Africa – with significant implications for adolescent mothers’ mental health and preparedness for parenthood.^9^

The risks from early motherhood are also layered with other social, economic, and epidemiological challenges. In many parts of sub-Saharan Africa, adolescent girls are disproportionately affected by HIV, with girls representing 80% of new HIV infections occurring among 15- to 19-year-olds.^10^ Rates of perinatal HIV acquisition and mortality among infants born to adolescent mothers in LMICs are higher the younger the mother is, a relationship which may be only partially attenuated by health care-seeking.^11^ Multi-country analyses of Demographic and Health Survey data from 10 sub-Saharan African countries found strong quantitative associations between early motherhood and living with HIV.^12^ In regions where HIV incidence in this group is persistently high, young women may learn of new HIV diagnoses as they attend antenatal visits, and are thus navigating two complex, life-changing transitions simultaneously.^12,13^

Importantly, adolescent mothers may also confront structural barriers that complicate their developmental, social, and economic transitions to adulthood and motherhood.^14,15^ Experiences of stigmatisation and isolation are common, often linked to gender norms about adolescent girls’ sexuality, and family and community attitudes towards virginity, marriage, and childcare.^16^ While family members can act as important sources of support during this time, family relationships can also create additional stress if they are characterised by discord or blame. Some of the same factors that drive early pregnancy and HIV infection may pose further barriers to service access; these include poverty, experiences of gender-based violence, and social and gender norms that inhibit conversations around sexual activity, condom use, and care-seeking for sexual and reproductive health (SRH) needs.^2,17^ Consequently, pregnant and postpartum adolescents may struggle in practice to access essential services and support at a time when they need it most. These experiences can also become entrenched in cycles: half of girls with a first birth between ages 15 and 17 have a second child before turning 20.^6^

Adolescent pregnancy, early motherhood, and HIV can be conceptualised as a syndemic, as these experiences interact with socioeconomic and environmental factors to increase the burden of disease at individual and population level.^18^ The COVID-19 pandemic has added a new, complicating dimension to these issues. Social and health system responses to COVID-19 resulted in significant service interruptions, including access to contraceptive services.^19^ Restrictions in access to routine health care and extended periods of isolation and lockdown have been reported as having negative effects on the health and wellbeing of adolescent girls, including mothers.^20–22^ Furthermore, many of the well-documented drivers of HIV acquisition in adolescent girls and young women – including food insecurity, lack of accessing to schooling, unemployment, and orphanhood – have been accelerated by the pandemic. Many of the impacts of this cascade of negative outcomes, including on gender equity and service access, will only be seen fully in the coming years.^23,24^

## Methodology

A growing body of evidence looks at the maternal health outcomes of adolescents in LMIC contexts and HIV-endemic settings. Our team’s previous scoping review, published in 2020, found 27 peer-reviewed publications that reported health-related data on adolescent mothers affected by HIV, and profiled nine interventions that aimed to support their health and wellbeing.^25^

However, this review also identified a notable evidence gap surrounding the normative, legal, and policy environments that shape the health outcomes of adolescent mothers and their children. In light of the complex vulnerabilities they are facing, pregnant and postpartum adolescents need safe and supportive policy environments and legal provisions conducive to the realisation of their human rights, especially the right to health, to experience nurtured transitions to motherhood. Conducive policies, laws, and human-centred services are critical to ensure that these young women and their children are well-supported at a pivotal life stage,^26^ yet little attention has been paid to this critical gap.

Building on our previously published scoping review, this paper highlights three core normative, legal, and policy areas affecting the health and wellbeing of adolescent mothers and their children in HIV-endemic settings. We integrate ongoing primary and secondary research in each area, combining primary research identified in our literature searches with findings from more recent hand-searched reviews and emerging findings from collaborators and our own work. We subsequently set out priorities for further research and intervention.

## Framing the contexts of violence and inequitable norms linked to early motherhood

A key issue is acknowledging the contexts of violence and gender inequity in which many early pregnancies occur. For many adolescent girls, contexts of violence precede and lead to early motherhood, and these conditions also continue during pregnancy and after they become mothers.^27^

Evidence from the Violence Against Children and Youth Surveys (VACS), captured from multiple countries over the past 15 years, has found that significant proportions of young women in sub-Saharan Africa who reported a first sexual experience before the age of 18 had experienced pressured or forced sex (Table 1). These numbers range from 8.9% in Kenya (2019)^28^ and 9.8% in Botswana (2019)^29^ to 16.9% in Zimbabwe (2017).^30^ Studies from South Africa and Zambia have found that adolescent girls and young women who experience sexual violence are more likely to experience an unintended pregnancy.^31,32^ Additionally, 23.2% of adolescent mothers in a large South African cohort had their first child before the age of 16, the legal age of consent, with a higher proportion of HIV-uninfected mothers giving birth by the age of 16.^33^ A review-of-reviews by Grose et al. further details the extent to which experiences of diverse forms of gender-based violence increase the risk of poor SRH outcomes for young women in LMICs, including more sexual partners, HIV and sexually transmitted infections, unwanted and unplanned pregnancies, and abortion.^34^ Legal requirements for reporting violence are often complex and can stigmatise survivors further; socially, adolescent girls and young women may be discouraged from reporting their experiences due to high levels of stigma, self-blame, and negative stereotypes about young women’s sexuality.^35,36^ Furthermore, these issues can be especially fraught when violence happens in the family context.

Linked to these experiences is child, early, and forced marriage. The most recent data from UNICEF indicate that in sub-Saharan Africa, 35% of girls were married by age 18, and 11% by age 15.^37^ This varies substantially by country setting; for example, data from March 2020 show that 76% of young women in Niger were married before age 18,^38^ and in Eastern and Southern Africa, 50 million girls and women who are alive today were married before their 18th birthday.^6^ Adolescent girls who are married before age 18 are more likely than their unmarried peers to initiate early childbearing, have lower socioeconomic status, and not complete secondary school.^39,40^ Importantly, child marriage itself may not be the primary driver for early motherhood, but may enable other risk factors for early pregnancy to become more entrenched, including age-disparate relationships, limited access to healthcare services, unprotected sex, and intimate partner violence.^41,42^ In sub-Saharan Africa, sexual activity, unplanned pregnancy, and school dropout often precede child marriage; thus, marriage may be linked to wider gender inequalities and cultural beliefs about premarital sex and childbearing, norms around family reputation, and lack of timely access to SRH services including safe abortion.^43^ Age-disparate relationships have been consistently documented as drivers of HIV acquisition in young women,^44^ and having a partner more than five years older is associated with higher risk of repeated pregnancy.^45^ Early marriage often increases the frequency and likelihood of unprotected sex, especially in contexts where women have limited negotiating power or skills, increasing HIV risk as well as leading to pregnancy.^14,46^ Girls who are recently married may also be expected to “prove” their fertility, leading to early conception and childbearing; a recent analysis showed that 56.1% of girls in child marriages had a child within the first year of marriage.^47^

Being married also shapes adolescents’ experiences of motherhood in significant ways. Even though married adolescent girls have a greater likelihood of experiencing early motherhood, they also face certain roadblocks to health and social service access. Contradictions among formal and customary laws in relation to definition of child marriage can greatly affect how adolescent girls are recognised in the legal system and granted protections by state and non-state actors.^48^ In some countries, a large proportion of child marriages are not officially registered,^49^ complicating girls’ access to education and/or health services without the consent of a husband or guardian, especially where age of consent to marriage and access to services differ. Distinguishing between age of consent to sexual activity and age of marriage is also critical; as countries have raised the legal age of marriage to 18, with no exception, there have been attempts to also raise the age of consent to sexual activity, which would likely further restrict adolescents’ agency and ability to access SRH services, and do not take into consideration the evolving capacity and the best interests of the child.^50^

On a broader level, inequitable gender norms place adolescent girls at further risk for exploitation and poor health, especially related to early pregnancy, repeat childbearing, and HIV.^51,52^ A study of postpartum mothers in South Africa found that adolescent mothers had more power-inequitable relationships and higher perception that their partners were engaging in concurrent relationships.^53^ Analyses from another South African cohort study found that more than half of adolescent mothers had had their first child with a partner five or more years older, with only 16% knowing the HIV status of their child’s father. Service provision models need to be more specifically attuned to these realities, bridging considerations of safeguarding with legal reporting requirements for a highly vulnerable population.

**Table 1:** Data on key relevant indicators from 13 priority high HIV burden countries for adolescent girls and young women^a^

Country	HIV prevalence (%), 15–24 years, girls^b^	Weighted ever-pregnant prevalence (%), 10–19 years^c^	Early marriage rate (% married by 18 years of age), girls^d^	First sex (<18 years) was pressured, forced, or unwanted, (%), girls^e,f^	Upper secondary school completion rate (%), girls^g^	Lower secondary school completion rate (%), girls^6^
Botswana	5.2	n/a	n/a	9.8	66	92
Cameroon	1.2	n/a	29.8	n/a	21	43
Eswatini	12.4	23	5.3	7.2	33	54
Lesotho	7.1	19	16.4	16.8 (Maseru) 22 (Berea)	37	55
Kenya	1.9	n/a	22.9	8.9	38	69
Malawi	2.7	30	42.1	37.7	13	21
Mozambique	n/a	38	52.9	4 (Gaza) 4.9 (Zambezia)	5	11
Namibia	7.1	25	6.9	6 (Khomas)4.5 (Oshikoto) 3.5 (Zambezi)	39	62
South Africa	10.2	19	3.6	8.2	52	91
Tanzania	1.9	17	30.5	29.1 (Tanzania) 9.6 (Zanzibar)	27	27
Uganda	2.7	n/a	34.0	20.4	15	23
Zambia	5.1	28	29.0	26.2	27	50
Zimbabwe	5.1	22	33.7	16.9	14	53

^a^ Technical Brief: HIV Programming for Adolescent Girls and Young Women in High-HIV Burden Settings, The Global Fund; 2020. https://www.theglobalfund.org/media/4576/core_adolescentgirlsandyoungwomen_technicalbrief_en.pdf.

^b^ Spectrum data, UNAIDS, 2022. https://naomi-spectrum.unaids.org.

^c^ Yah CS, Ndlovu S, Kutywayo A, Naidoo N, Mahuma T, Mullick S. The prevalence of pregnancy among adolescent girls and young women across the Southern African development community economic hub: A systematic review and meta-analysis. Health Promot Perspect. 2020;10(4):325–337. doi: 10.34172/hpp.2020.51.

^d^ Child marriage data, UNICEF, May 2022. https://data.unicef.org/resources/dataset/child-marriage/.

^e^ Violence Against Children and Youth Survey Reports, https://www.togetherforgirls.org/en/resources?type=4708&p=1.

^f^ Richter L, Norris SA, Ramjith J. Early sexual debut: voluntary or coerced? Evidence from longitudinal data in South Africa-the Birth to Twenty Plus study. South African Med J. 2015;105(4):304–307.

^g^ Secondary education data, UNICEF, June 2022.

### Exploring barriers to accessing quality and integrated sexual and reproductive health services and information

A second key issue surrounding early motherhood is adolescent girls’ ability to access quality, people-centred, and integrated SRH services and information. Adolescent girls and young women can struggle to access adolescent- and youth-responsive health services, especially when they are seeking contraceptives. This issue is particularly important when the legal age of consent – to health services, sexual activity, and marriage – is misaligned with the age of first childbearing, which often varies by country. In other words, it is critical to assess when adolescent girls are requiring these services, and link this to when they are able to access them on their own. Alignment should also be ideally linked with evidence-informed standards. In South Africa, for instance, the age of consent to sex is 16, but a minor girl of any age is allowed to consent to terminate her own pregnancy without additional consent and a 12-year-old can consent alone to an HIV test.^54–56^ In other countries, however, access for both pregnancy prevention and core SRH services is less defined.

Pregnant adolescents and adolescent mothers, including both married and non-married girls and young women, often face discrimination and mistreatment when they seek health care.^57,58^ Girls under 18 years of age may be treated as minors or “children having children,” discouraged or barred from accessing preventative health services for sexual and reproductive health (including contraception and safe abortion, where legal) as well as HIV (including testing, counselling, and treatment). This response is likelier in settings where these services are not integrated.^59,60^ In parallel, while rates of postpartum contraception immediately post-birth tend to be high, contraceptive use tends to drop off within about a year. Rates of current contraception use in a study of adolescent mothers – 60% of whom had children aged one year or older – were less than two-thirds.^9^ For the most marginalised adolescent girls, including those involved in sex work, those heading households and/or caring for younger siblings, and victims of child sexual abuse and exploitation, these challenges and stigmatising experiences are likely to be even greater.^61^

In some countries, adolescent mothers become automatically emancipated, legally able to access services for themselves and their children without a guardian.^62^ For adolescent mothers and married adolescents, national guidelines may encourage HIV testing at younger ages than for the general adolescent population.^63^ Ten Eastern and Southern African countries follow international best practice with policies enabling adolescents ages 12–16 to access HIV testing without parental consent.^26^ However, in many sub-Saharan African countries, policy frameworks and enacted legislation may conflict, impeding adolescents’ access to much-needed health services. Omissions in policies and laws can also enable exclusion. Without a clearly defined age of consent for health services in national legal frameworks, interpretation relies upon health providers’ discretion – which can be especially damaging for childbearing adolescents who face stigma while also requiring more comprehensive services.^64,65^

Nestled within these legal strictures are social factors that influence adolescent girls’ ability, or willingness, to seek care. Adolescent girls may have limited access to funds to travel to clinics or to pay for certain services in settings where care is not freely available.^66^ Internalised stigma, as well as stigmatising attitudes by health providers and/or family members, may also discourage timely care-seeking. Even in settings where progressive policies exist – for instance, around termination of pregnancy from a young age – adolescent girls may be actively dissuaded from accessing these services. Integrating HIV and SRH services is critical in HIV-endemic areas, and can enable better detection of new pregnancies and new HIV infections; this is especially important given shifts in multi-month ART dispensing practices and ongoing SRH-related stigma for adolescent mothers.

Beyond reforming legislation and reframing national policies to remove barriers to access, these considerations form part of a broader conversation about how to support adolescent girls in settings where their bodily and social autonomy is generally restricted. In tandem with making services more available, acceptable, and adolescent-responsive, laws and policies to expand access to service provision must respond to culturally and socially rooted norms that accompany adolescent girls throughout their developmental trajectories. Analyses from a large cohort of South African adolescents highlighted how adolescent-responsive health services (i.e. where adolescents reported not feeling judged about skipping medication, appointments, or having sex) were significantly associated with greater odds of HIV retention in care and safe sex among adolescents living with HIV, especially adolescent girls and young women.^67^ Existing global standards for adolescent-friendly health services are aspirational but their application in low-resource settings remains limited.^68–70^ Therefore, evidence is urgently needed to understand which healthcare services features must be prioritised to maximise adolescent outcomes.

## Identifying bottlenecks in access to essential social and structural supports

A third and final key issue focuses on the essential social and structural support available for adolescent mothers, both during their pregnancies and after they give birth. Evidence suggests that transitions back to secondary education and access to integrated social protection and services are critical for adolescent mothers, yet are currently poorly or unevenly implemented.^25^

Policies that underpin continued access to education during pregnancy and early motherhood are essential. Across sub-Saharan Africa, policies for pregnant and postpartum adolescents range from restrictive (such as immediate school expulsion upon confirmation of pregnancy)^71^ to protective and transformative (providing a comprehensive package of pre- and post-natal support for both mother and child).^72^ In some settings, adolescent mothers may have to carry the blame alone from family members and community members for their pregnancies, or be castigated for negatively influencing peers.^73^ Even where progressive and inclusive policies exist to support re-enrolment after childbirth, they may not be consistently applied or implemented.^74^ Where national policies are absent, school administrators, staff, or parent boards may dictate the terms of re-enrolment. Girls may be obliged to apply to a different school, enrol in evening programmes, or undergo a minimum “waiting period” before re-enrolling.^71^ Opportunities to link adolescent mothers to vocational training or alternative educational pathways are also limited. Consequently, many adolescent mothers struggle to return to school, even in contexts where reintegration is endorsed from a legal perspective.^75,76^ Gender inequity is clear in these cases, as adolescent fathers face almost none of the same discrimination or barriers to their schooling.

There is growing evidence that social protection, particularly social assistance in the form of cash and food transfers, can help address educational, social, and health service barriers. Models of pregnancy support for expectant women of all ages, in the form of cash transfers or other social transfers (such as food parcels), have been implemented across at least 27 countries.^77^ However, limited programmes exist to support adolescent mothers. Development agencies are increasingly considering how social protection investments can be leveraged to promote more age- and gender-responsive outcomes, including “cash plus” interventions which combine household receipt of social protection with SRH and life skills support.^78^ There are prevailing stigmatising attitudes about how government support might incentivise young women to become pregnant, although these notions have been largely debunked by evidence.^79^ Child care provision is another structural barrier to educational return, despite its clear utility both in supporting the mother and in providing quality care for the child. There is increasing recognition that instrumental support at the early stages of adolescent mothers’ trajectories can keep them on track to reach health, educational, and economic goals, but more specific actions may be needed by members of civil society as well as global stakeholders to increase political will and the investment case for these types of support.

## Emerging evidence to support appropriate responses

Because of the complexities and inconsistences that adolescent mothers navigate within normative, legal, and policy environments, more robust responses are needed to support adolescent mothers to access services to protect their health, education, and safety. These responses should ideally be rights-based, comprehensive, multi-level, and multisectoral in nature. Notably, specific responses may be able to address one or more of the areas discussed above.

Emerging evidence supports **three specific responses** that could be particularly far-reaching, for both married and non-married adolescent girls and young women: (1) responding to and counteracting barriers to SRH and HIV service integration; (2) ensuring implementation of return-to-school policies; and (3) better scaling-up of age- and gender-responsive social protection in context-specific ways.

**First, harmonising according to their “function”, age-of-consent provisions to access SRH services and HIV testing would enable adolescent mothers to access necessary preventive services including contraceptives to prevent second pregnancies and HIV care or treatment, while also ensuring their children are healthy and their postpartum care needs are met**. Importantly, minimum ages of consent must not be the same across laws for marriage, sexual activity, and access to integrated HIV and SRH services.^80^ However, harmonising these laws would remove barriers to SRH and HIV service integration and facilitate more fluid and less stigmatising access to essential care.

**Second, standardising return-to-school policies across contexts, so that they are implemented and accountable in a way that maintains the dignity of pregnant adolescents and adolescent mothers and prioritises their education, is also essential**. Knowledge of school policies may be one way that pregnant adolescents and adolescent mothers can push back against discriminatory treatment on an individual level.^74^ However, beyond non-discriminatory policies, multiple forms of tangible support can further ease these transitions. Implementing policies through evidence-informed packages is a critical next step where the legal and regulatory framework is supportive. Recent data have found that adolescent mothers are likelier to return to school if they can access and afford child care^81^ and if they receive support from their families as well as the school itself.^82^ On a broader level, delivering evidence-based SRH information in schools may support subtler institutional shifts in norms and make education about sex, sexuality, and pregnancy more readily available.

**This evidence supports a third and final response – extending and adapting existing social protection provisions to be more inclusive of adolescent girls including adolescent mothers – which could provide transformative support at critical moments in their developmental trajectories, and those of their children**. Many countries have existing social protection measures for children; in South Africa, the Child Support Grant could be extended into the pregnancy period, beginning approximately 9–12 months earlier than it currently does for most women.^83^ Initiation onto this grant could be linked with early antenatal visits and school-based service provision. While this policy is maternity-specific rather than adolescent-specific, it has the potential to transform outcomes for more vulnerable mothers and their children, which may include adolescent mothers and those affected by HIV. Numerous cases for this investment have been made, with broadly recognised potential benefits such as reductions in child marriage and better educational attainment.^84^

More broadly, social assistance through continual cash or food provisions may be able to reduce the likelihood of transactional sex, sexual violence, and poor mental health, contributing to pregnancy prevention or supporting better-timed second pregnancies.^85^ Integrated social support may also be particularly effective. Although there is mixed evidence about the impact of cash transfers alone on delaying adolescent childbearing,^86,87^ integrated social protection approaches to reducing vulnerability, such as combining cash transfers with care or other provisions, may hold more promise.^88^ *Ujana Salaama*, one such programme delivered to rural Tanzanian youth, combines cash transfers with in-person training, mentoring, and linkages to health services.^89^ These combination models are important to probe for adolescent girls and young women who are already experiencing early motherhood. Structural supports might also be more broadly conceived. More tailored programmes for HIV-affected populations, such as peer mentor mothers or trained peer supporters, may be able to provide non-judgmental care to improve psychosocial and physical health outcomes among adolescent mothers.^25,66^ Additional socioeconomic programming can also be effectively adapted across broader populations of adolescent girls and young women in HIV-endemic communities, providing life skills, social capital, and mentorship in HIV-sensitive ways.^90^

## Discussion and conclusions

Adolescent mothers in HIV-endemic settings face a distinct set of interlinked adversities as they transition to adulthood. While many girls who are married or have children have socially transitioned to adulthood, they are still treated as minors in national law and policy frameworks. Experiences of violence and gender inequity are often in the background of early pregnancies, but adolescents with these experiences may be stigmatised, neglected, or overlooked in service provision settings. Pregnant and parenting adolescents caring for themselves and their children may be barred from accessing critical health services, contributing to risk of HIV infection, onwards HIV transmission, and repeated unintended pregnancies. Adolescent mothers experiencing a premature end to their education may be forced to forego valuable social relationships and later be excluded from formal employment. These mothers may face additional economic hardships, which can lead to increased HIV-related risk behaviours.

Importantly, these negative consequences can become amplified in the context of shocks such as war and humanitarian settings, climate crises, and pandemics.^3^ The COVID-19 pandemic has compounded the challenges that adolescent mothers face. In addition to increased estimates of unintended pregnancies to adolescents and young women, UNFPA estimates from March 2021 forecast that there will be as many as 10 million more child marriages than previously expected in the coming decade.^91^ Interruptions in care related to COVID-19 also reduced care-seeking behaviours overall, disrupting access to family planning and HIV testing services, and creating bottlenecks in available services.^23,92,93^ In addition, widespread school closures imperilled adolescent girls’ learning trajectories and interrupted access to preventive school-based SRH programming, increasing their risk of first or repeated unintended pregnancies.^20,22,94^ Along with increases in household economic precarity, the ongoing ramifications of the pandemic may pose ongoing challenges for vulnerable families who can no longer afford to send their daughters to school, perpetuating cycles of risk. Ensuing early, unplanned motherhood may put young mothers and their children in vulnerable positions, requiring short-term relief as well as long-term developmental support.

Beyond considering programmatic, legal, and policy responses, our review also highlights several key research gaps where further work could inform our understanding of the needs of adolescent mothers in HIV-endemic settings. More evidence is needed to characterise and address adolescent mothers’ experiences of violence, and the social scripts that perpetuate gender inequity and harmful norms. Given the ethical complexity that often makes it difficult to conduct research with adolescent mothers experiencing or exposed to violence, more inter-disciplinary work and an ongoing investment by funding agencies to prioritise this group is required. Additionally, while emerging research is pointing to promising interventions that can mitigate health and social risks facing adolescent mothers and their children, it will be critical to isolate services and combinations of services that can improve multiple outcomes at the same time, in light of competing funding priorities. Further research could also provide more nuanced understanding of how tailoring health services to adolescents’ needs, and at critical life events, could improve outcomes linked to health, wellbeing, and socioeconomic stability.

A number of emerging service provision models may provide a context for these research areas: peer-based psychosocial support for adolescent mothers, delivered by trained youth who are supported by clinic-based or other staff, may support in shifting norms and reducing stigma.^66^ Sensitive care provision by health care workers can similarly bridge gaps in policies that are lagging, by offering adolescent mothers interactions characterised by dignity and high-quality care.

On a longer time-horizon, coordinated sensitisation and advocacy are required on multiple levels to amend discriminatory laws and policies and eliminate harmful practices, while upholding protective systems and structures and fostering access to services by adolescents. Promoting more equitable, gender-transformative, rights-focused laws and policies can help to protect the health, wellbeing, and rights of adolescent mothers and their children. These provisions need to be accompanied, however, by political will, law enforcement measures, financial resources, infrastructure, and accountability mechanisms. From a normative perspective, changes may proceed more gradually. Because many forms of gender and structural inequality facing adolescent girls and young women are deeply entrenched and interconnected, shifting them may require a more nuanced, coordinated approach across sectors.^95^

This work has several limitations; while we built on gaps identified through publications in our prior review, it is possible that other salient normative and policy issues may be missing from this analysis. Because this work was not based on a systematic search, relevant research may have been unintentionally omitted. While most literature on adolescent girls and young women in high-HIV burden settings centres on sub-Saharan African countries, there may be additional work examining the experiences of girls and young women in other settings that would add to our findings.

Importantly, a steady increase in the realisation of sexual and reproductive health and rights for adolescent girls and young women, including mothers, is critical – but also tenuous. Safeguarding these rights depends on reliable funding for programming and continued support from international as well as local stakeholders. Considering ongoing social, economic, and humanitarian crises, action and attention revolving around these key normative, legal, and policy considerations are critical to promote maternal and child health across the life course and safeguard future generations.
